# Influence of Different Plant Extracts on CYP-Mediated Skatole and Indole Degradation in Pigs

**DOI:** 10.3390/ani14060888

**Published:** 2024-03-13

**Authors:** Philipp Marro, Raffael Wesoly, Volker Stefanski

**Affiliations:** 1 Department of Behavioral Physiology of Livestock, University of Hohenheim, 70599 Stuttgart, Germany; 2 German Genetic Schweinezuchtverband Baden-Württemberg e.V., 70599 Stuttgart, Germany

**Keywords:** pig, CYP2E1, CYP2A, garlic, oregano, *Schisandra chinensis*

## Abstract

**Simple Summary:**

One of the main substances responsible for the unpleasant odor in boar meat is skatole, which is created by the breakdown of tryptophan by bacteria in the hindgut of pigs. This study aimed to assess the impact of three different plant extracts on skatole levels in the blood and adipose tissue of pigs, while elucidating their relationship with the hepatic skatole metabolism. *Origanum vulgare* essential oil and *Schisandra chinensis* extracts were chosen for their potential to mitigate intestinal skatole formation or enhance hepatic skatole degradation, thereby reducing its accumulation in adipose tissue. Garlic essential oil was investigated due to its established capacity to elevate skatole concentrations in adipose tissue. The findings revealed that garlic essential oil significantly influenced skatole accumulation by impeding hepatic degradation, whereas both oregano essential oil and *Schisandra chinensis* extracts had no discernible impact on skatole metabolism or its adipose tissue concentrations.

**Abstract:**

One of the primary substances responsible for the unpleasant odor in boar meat is skatole. Enzymes belonging to the cytochrome P450 (CYP) family play a pivotal role in the hepatic clearance of skatole. This study aimed to investigate the impact of oregano essential oil (OEO), *Schisandra chinensis* extract (SC), and garlic essential oil (GEO) on hepatic CYP2E1 and CYP2A activity in pigs. In three consecutive trials, cannulated castrated male pigs were provided with a diet containing 0.2–0.3% of one of these plant extracts. Following a 14-day feeding period, the animals were slaughtered, and liver and fat samples were collected. The findings indicate that the activities of CYP2E1 were unaffected by any treatment. However, GEO treatment demonstrated a significant reduction in CYP2A activity (*p* < 0.05). Pigs treated with GEO also exhibited a notable increase in skatole concentrations in both plasma and adipose tissue. In contrast, animals fed SC displayed elevated skatole concentrations in plasma but not in fat tissue. OEO did not influence skatole concentrations in either blood or fat. Furthermore, the study revealed that a supplementation of 6 g GEO per animal per day induced a significant increase in skatole concentrations in blood plasma within 24 h.

## 1. Introduction

Surgical castration of pigs without pain relief is at present regarded unacceptable in many countries. Traditionally, the castration of entire male pigs has been conducted to prevent boar taint, which has been primarily attributed to the accumulation of androstenone, skatole, and other minor contributing indoles in the adipose tissue of intact male pigs [1,2,3]. Alternative methods to traditional surgical castration employed to mitigate boar taint, in particular androstenone, include procedures such as surgical castration with anesthesia and analgesia, and the fattening of boar. Androstenone is a testicular steroid and serves as a pheromone in porcine species. Its synthesis is intricately linked to the production of testicular hormones and, consequently, to pubertal development [4]. Skatole (3-methylindole) and other indoles are generated in the large intestine of pigs and various other species through microbial degradation of the amino acid L-tryptophan [5].

Both skatole and indole are absorbed by the gut, with most of them undergoing rapid metabolism in the liver. However, due to the lipophilic nature of skatole, a certain percentage is also deposited and accumulates in adipose tissue [6]. Several studies describe the involvement of enzymes of the cytochrome P(450) CYP family in skatole degradation, demonstrating that the hepatic clearance of skatole is strongly dependent on the activity of the key enzymes CYP2E1 and CYP2A [7,8,9]. Prior investigations into boar taint and its correlation with porcine CYPs have highlighted the significance of endocrine status and gender in influencing the expression of CYP2E1 [10]. Further studies [11,12] have demonstrated that the presence of androstenone, along with high testosterone levels, contributes to the down-regulation of CYP2E1 expression, leading to diminished skatole degradation. In contrast, surgical castration and immunocastration have been found to result in higher activities of CYP2E1, CYP2A, and CYP1A [13].

Given the important role of P(450) CYP in pharmacology, especially in drug clearance, extensive research has been conducted to uncover potential implications of substances affecting this enzyme complex for drug metabolism. Numerous studies have reported that phytochemicals present in dietary compounds can influence various CYPs [14]. Consequently, there is a growing interest in considering a specific diet as an access point for CYP-mediated degradation. Rasmussen et al. [13] and Čandek-Potokar et al. [15] have suggested that diet could be a potential factor in enhancing hepatic clearance by supplementing plant extracts and their respective bioactive constitutes. Several studies have explored the role of phytochemicals, such as varying concentrations of polyphenols like tannins, flavonoids, and lignins (ranging from 1% to 10% per meal), when used as dietary additives [16].

*Schisandra chinensis* is commonly used in traditional Chinese medicine (TCM), owing to its well-known hepatoprotective properties [17]. The fruits, leaves, and stems of *Schisandra chinensis* contain dibenzo[a,c]cyclooctadiene lignans and are thus suggested to have bioactive properties [17]. An in vitro study examining the hepatic effects of *Schisandra chinensis* confirmed its role in activating Nrf-2 on HepG2 cell lines [18]. Nrf-2 activation is known to mediate the activation of target genes involved in phase I and phase II liver metabolism [19]. Further studies have reported an induction of CYP2E1 expression following the oral administration of *Schisandra chinensis* in mouse and rat models [20,21].

In contrast, garlic essential oil (GEO) has the opposite effect on CYP2E1. Studies conducted in rats have demonstrated that garlic is capable of inducing a decline in P450(CYP)2E1 activity, attributed to the interaction of diallyl sulphide with the protein structure of CYP [22,23]. In order to obtain garlic-flavored pork meat, Leong et al. [24] introduced GEO into the diet of growing pigs. This intervention resulted in higher concentrations of skatole in adipose tissue, assuming a significant reduction in CYP activity. Such considerations become crucial when defining a standardized challenge for testing different genotypes for their susceptibility to skatole accumulation in adipose tissue [25].

*Origanum vulgare* comprises the bioactive compounds thymol and carvacrol, both recognized for their antimicrobial properties [26]. Studies conducted on human liver microsomes have revealed inhibitory effects against CYP3A4. However, information regarding potential effects on porcine CYP2E1 and CYP2A is currently limited.

The primary objective of the present study was to investigate whether concentrations of skatole and indole in blood plasma and adipose tissue can be modulated by the addition of plant extracts and oils in the diet and whether such effects could be explained by alterations in hepatic P(450) CYP 2E1 and CYP2A activity. Specifically, *Schisandra chinensis* extracts (SC) and oregano essential oil (OEO) were assessed as dietary additives in order to examine their potential in reducing skatole accumulation in adipose tissue, while GEO was investigated for its potential to increase skatole levels in adipose tissue. Our hypothesis posited that a supplementation of SC and OEO in the diet would enhance hepatic enzyme activity, thus preventing skatole deposition in adipose tissue. Conversely, in the case of GEO, we hypothesized that CYP2E1 in pigs would be affected in a similar way to that observed in rats and mice, and thus aimed to evaluate its actual influence on skatole degradation. Our study intended to clarify whether these plant extracts can be used as a rapid and efficient tool for in vivo identification of genotypes that exhibit a lack of responsiveness to GEO. Such non-responsiveness may potentially indicate the presence of polymorphisms in the coding genes of CYP2E1 and CYP2A attributable to elevated expression rates.

## 2. Materials and Methods

### 2.1. Animals, Experimental Design, and Sampling

Our study was performed at the experimental unit of the Department of Behavioral Physiology of Livestock at the University of Hohenheim (Stuttgart, Germany). All experiments were approved by the ethics committee for animal experiments by the regional authority (Regional Council, Stuttgart, Germany; approval number V307/13TH). All data presented here are part of a comprehensive experiment examining the impact of plant additives on both skatole formation and degradation, using the same animals, experimental design, and methodology (Marro, Wesoly & Stefanski, submitted).

A total of 36 castrated male pigs (German Landrace × Piètrain) were studied in three consecutive trials. The animals (initial body weight: 90 ± 5 kg) were housed individually in pens of 5.3 m^2^ that enabled visual and tactile contact with other pigs, cushioned with dust-free wood shavings. The animals were kept under a light regime of 12 h/12 h (light/dark), with free access to water. A concentrated standard feed (1.5 kg/meal, metabolizable energy: 14 MJ/kg; Appendix A) was provided twice per day.

Each trial consisted of two periods: In period 1 (experimental days 1–14), all pigs were provided a standard diet (Appendix A). Given that castrates intrinsically produce low levels of skatole, 12.1% of dried brewer’s yeast was given in accordance with the standard ratio to assure a high availability of tryptophan for skatole-producing bacteria and, consequently, to stimulate natural skatole production. During period 2 (experimental days 15–29), all pigs, excluding the control group (CON), were fed the standard diet along with one of the plant additives as a top dressing. This resulted in four groups: CON (*n* = 12), SC (*n* = 8), OEO (*n* = 10), and GEO (*n* = 6). Due to the intense odor of the plant additives, the animals were habituated to the smell. Moreover, to avoid potential side effects due to the intense and different scents, the study was conducted in successive trials, each involving the supplementation of a single plant additive.

For stress-free and frequent blood sampling, indwelling jugular vein catheters were implanted in all pigs three weeks prior to the start of the experiment. Blood samples were collected daily at 08:00 h from the catheters for analysis of skatole and indole concentrations. During the first experimental period (days 1–14, without plant additives in the diet), there were 9 catheter failures. In the second experimental period (days 15–29, with plant additives in the diet), one catheter failure occurred (in the SC group), rendering daily blood sampling unfeasible. This resulted in an altered number of experimental animals available for evaluating the trajectory of skatole and indole concentrations over the entire experimental period. The actual numbers of animals used for analysis, therefore, were as follows: CON (*n* = 3), SC (*n* = 7), OEO (*n* = 10), GEO (*n* = 6).

In order to monitor skatole and indole concentrations in adipose tissue during the experimental periods, punch biopsies of back fat were conducted at weekly intervals (days 0, 7, 14, and 21), in accordance with [27,28]. Due to a high stress response, fat samples could not be obtained from 3 animals at one respective sampling date.

At the end of the experiment, all pigs were slaughtered in order to obtain a final sample of liver and adipose tissue from the back.

### 2.2. Dietary Additives

Oregano essential oil (OEO) (DOSTO^®^ Konzentrat 500, powder, Dostofarm^®^ (Westerstede, Germany)) and *Schisandra chinensis* extract (SC) (Xian Yuensun Biological Technology Co., Ltd., Xi’an, China) were each added at a final concentration of 0.3% to the diet. Garlic essential oil (GEO, 80X, NS; KALSEC^®^ Europe Ltd., Leicester, UK) was added at a concentration of 0.2% (Table A1).

### 2.3. Analytical Methods

#### 2.3.1. Skatole and Indole Determination in Adipose Tissue

Measurements of skatole and indole were carried out via UHPLC (Dionex Ultimate 3000 RS and Dionex Ultimate 3000 RS pump) and a fluorescence detector, based on the protocol of Wesoly et al. [29]. In brief, 100 µL melted fat was dissolved in 1 mL hexane. Thereafter, a solvent distribution was carried out with acetonitrile–water (4:1). After mixing and centrifugation, the hexane phase was removed, and the remaining sample was measured via UHPLC with the florescence detector at an emission wavelength of 275 nm and an extinction wavelength of 352 nm.

Precision was determined by measuring skatole- and indole-spiked samples. Average recovery rates for skatole and indole were between 93% and 99%. Intra-assay and inter-assay variabilities were determined with biological samples for skatole and indole and were below 10% each.

#### 2.3.2. Skatole and Indole Determination in Blood Plasma

The concentrations of skatole and indole were determined according to the protocol of Wesoly et al. [29]. Briefly, skatole and indole were extracted from blood plasma (500 µL) with diethyl ether (2 mL). After mixing and centrifugation of the samples, the aqueous phase was frozen and the liquid supernatant was transferred into a vial with 500 µL eluent. After the evaporation of diethyl ether at 50 °C in a heating block, the concentrations in the remaining eluent were determined by UHPLC.

Precision was determined by measuring skatole- and indole-spiked blood samples. The mean recovery rate for skatole and indole was between 92% and 102% for samples spiked with 125 ng/mL and 250 ng/mL, respectively. Intra-assay and inter-assay variabilities were determined with biological samples for skatole and indole and were below 10% each.

#### 2.3.3. CYP Assays

The preparation of microsomes from liver samples was based on the protocol of [30]. Aliquots of the microsomal samples were stored at −80 °C until analysis. The quantification of microsomal protein was carried out with a commercial kit using bicinchoninic acid (Applichem GmbH, Darmstadt, Germany). Bovine serum albumin was used for the calibration curve.

CYP2E1 activity was determined photometrically by measuring the formation of p-nitrocatechol according to Gao et al. [31] with slight modifications as per Cheng et al. [32]. The final reaction mixture of 400 µL contained 0.4 mg/mL of microsomal protein, 0.5 mM 4-nitrophenol, 1 mM NADPH, and an incubation buffer (0.1 M PBS). After incubation for 60 min in a 37 °C water bath, the reaction was stopped by adding 100 µL 20% trichloracetic acid.

After vigorous mixing and subsequent centrifugation (10,000× *g* for 5 min), 500 µL of the supernatant was transferred into 250 µL of 2 M NaOH. After a further mixing step, the samples were transferred onto a 96-well plate (NUNC, Thermo Fisher Scientific, Waltham, MA, USA). Measurements were carried out at 530 nm on a plate reader (X8000, BioTek, Winooski, VT, USA). The calibration curve covered the range of 0.625 to 20 nmol nitrocatechol and was prepared in buffer after the addition of heat had inactivated the microsomal protein (0.4 mg/mL). The enzymatic activity was expressed in pmol/min*mg protein. Precision was determined by measuring nitrocatechol-spiked microsomal protein samples. However, due to technical limitations, two samples could not be analyzed. The mean recovery rate was 93%. Intra-assay variability was lower than 10%, and inter-assay variability was below 15%.

CYP2A activity was measured fluorimetrically by measuring coumarin 7-hydroxylation formation according to the protocol of Zamaratskaia et al. [33] with slight modifications. The final reaction mixture of 400 µL contained 0.4 mg/mL microsomal protein, 0.2 mM coumarin, 0.5 mM NADPH, and an incubation buffer. 4-methylumbelliferone (50 nmol) was used as an internal standard. The 60 min incubation in a 37 °C water bath was stopped by adding 100 µL 20% trichloracetic acid. After vigorous mixing and a subsequent centrifugation step (10,000× *g* for 5 min), the supernatant was transferred to a glass vial and measured by UHPLC. The enzymatic activity was expressed in pmol/min*mg protein. Precision was determined by measuring 7-hydroxycoumarin-spiked microsomal protein samples. The mean recovery rate was between 94% and 97%. Intra-assay and inter-assay variabilities were below 10%.

### 2.4. Statistical Analysis

Statistical analyses were carried out using the IBM SPSS Statistics Version 23 software (Armonc, NY, USA). All data were normalized. If residuals failed to pass the normality test (Shapiro–Wilk), *logarithm* and square *root transformations* were performed. In order to evaluate the effect of the different treatments on skatole and indole accumulation in adipose tissue on the day of slaughter (day 29), one-way ANOVAs (fixed effect: treatment and CYP activity) for each compound were used. In order to detect differences in the accumulation of skatole and indole in adipose tissue over the entire study, statistical evaluation was performed for each day with included and excluded fat samples: days 0, 7, 14, 21, and 29, by one-way ANOVAs (fixed effect: treatment). In the absence of a normal distribution of residuals, a non-parametric Kruskal–Wallis test was used. In order to evaluate whether a change in skatole and indole concentrations in plasma occurred between the control period (days 1–14) and the treatment period (days 15–28), a paired *t*-test was performed. In order to clarify if the feed additives led to a significant short-term response in plasma 24 h after the first feeding of herbal additives, the respective concentrations of skatole and indole were compared in the last sample before feeding the additive (day 15) and 24 h later (day 16; paired *t*-test).

Least square (LS) means were calculated for ANOVAs and considered significant when *p* < 0.05. Differences between groups were identified using Games–Howell or GT-2 (Hochberg) post hoc tests.

Correlations between CYP2E1 or CYP2A and skatole or indole in adipose tissue and plasma, respectively, were evaluated by the Spearman’s rank correlation coefficient. This evaluation was performed with the complete dataset, irrespective of the treatment group, (r); *p* < 0.05 was considered significant.

## 3. Results

### 3.1. Effects of the Plant Extracts on Skatole Concentrations in Blood Plasma

**Figure 1:**
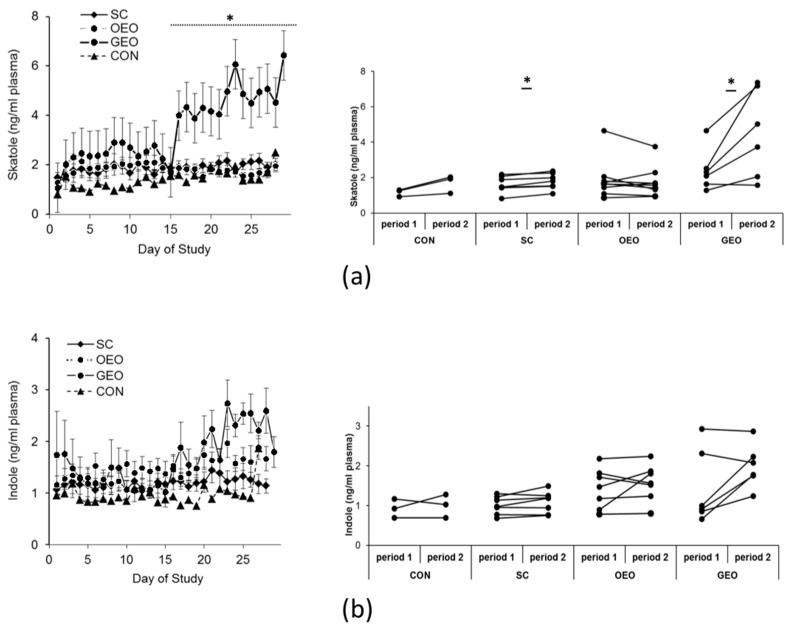
Means ± SEMs of plasma concentrations of skatole (**a**) and indole (**b**) throughout the experimental period. From day 1 to day 14 (period 1), all animals received the same standard diet. From day 15 to day 29 (period 2), the animals received one of the plant additives, *Schisandra chinensis* (SC; *n* = 7), oregano essential oil (OEO; *n* = 9), or garlic essential oil (GEO; *n* = 6), in addition to their standard diet, or received food without additives (CON; *n* = 3). ANOVA and paired *t*-test, * *p* < 0.05 is considered significant in comparison to CON.

Skatole and indole concentrations in plasma throughout the experimental period are depicted in Figure 1. The supplementation of GEO for 14 days resulted in a significant 83% increase in skatole levels in blood plasma (by 2 ng/mL on average; *p* < 0.05) compared to the control period (days 1–14). Administration of SC led to a modest 11% increase (*p* < 0.05), while the addition of OEO had no discernible effect on plasma skatole concentrations. One animal, however, exhibited no response at all (mean of period 1: 1.63 ng/mL, mean of period 2: 1.57 ng/mL; referred to as a “non-responder animal” in the following discussion) (Figure 1a). Nevertheless, animals receiving GEO as a dietary supplement displayed a notable increase in skatole concentrations within 8 h in plasma and within 36 h in back fat (Table 1) (*p* < 0.05). None of the plant additives had an impact on indole concentrations in the plasma (Figure 1b).

### 3.2. Effects of the Plant Extracts on Hepatic CYP Activity and Accumulation in Back Fat

**Figure 2:**
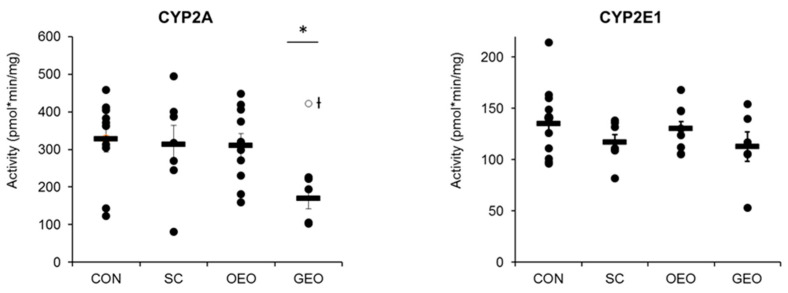
CYP activity (mean ± SEM) of the control (CON; *n* = 10), *Schisandra chinensis* extract (SC; *n* = 7), oregano essential oil (OEO; *n* = 9), and garlic essential oil (GEO; *n* = 5) groups after 14 days of supplementation; one-way ANOVA, * *p* < 0.05 is considered significant in comparison to CON; °Ɨ = non-responder and excluded from statistical evaluation.

Statistical analysis revealed that the level of CYP2E1 had a significant effect (*p* < 0.05) on skatole accumulation in back fat on day 29. Only GEO had an effect on CYP2A activity, resulting in a 48% reduction (*p* < 0.05) (with non-responders excluded) (Figure 2), indicating a clear impact on porcine hepatic enzyme activity. CYP2E1 activity was not affected by any of the plant additives.

**Figure 3:**
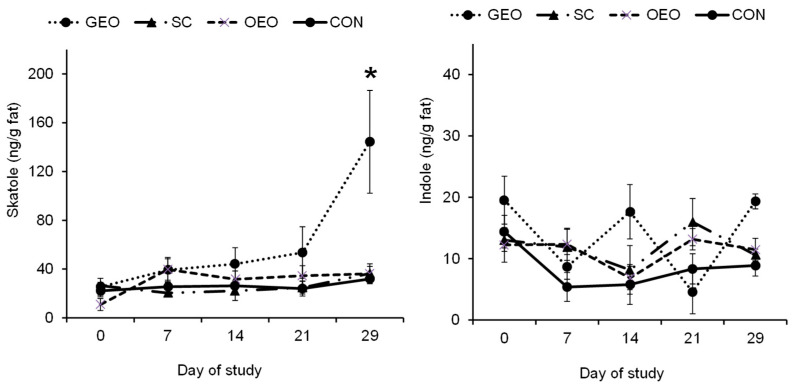
Skatole and indole concentrations in back fat (means ± SEMs) of control animals (CON; *n* = 10) and animals fed either *Schisandra chinensis* extract (SC; *n* = 7), oregano essential oil (OEO; *n* = 9), or garlic essential oil (GEO; *n* = 6); one-way ANOVA, * *p* < 0.05 is considered significant in comparison to CON.

The measurements of skatole in back fat are presented in Figure 3. Comparing the different sampling days (0, 7, 14, 21, and 29), a significant increase (*p* < 0.05) on day 29 was evident in the GEO group. The SC and OEO groups did not differ from the CON group with regard to accumulated skatole concentrations in adipose tissue. Moreover, no treatment-related effect on indole could be observed.

### 3.3. Relationship between CYP Activities, Skatole, and Indole in Plasma and Back Fat

#### 3.3.1. Relationship between CYP Activity, Skatole, and Indole Concentrations in Plasma and Back Fat at the End of the Study

The associations between skatole concentrations in plasma and back fat and the activity of CYP2E1 and CYP2A were examined independently for each treatment group and collectively for all animals within a unified dataset. Skatole concentrations in the back fat exhibited negative correlations with both CYP2E1 (r = −0.45, *p* < 0.01, *n* = 34) and CYP2A (r = −0.43, *p* < 0.01, *n* = 36). Consequently, the correlation of skatole in plasma on day 29 with CYP2E1 was negative (r = −0.48, *p* < 0.05, *n* = 20), as was the case for CYP2A (r = −0.41, NS; *p* = 0.06; *n* = 22), thereby affirming the significance of both CYP enzymes for plasma and adipose tissue concentrations of skatole. In contrast, indole demonstrated no significant association with either of the two CYP enzymes (*p* < 0.69).

#### 3.3.2. Relationship between CYP Activity, Skatole, and Indole Concentrations in Plasma Back Fat throughout the Experimental Period

The relationship between CYP activity, skatole, and indole concentrations in back fat and plasma was examined, utilizing data obtained from the weekly performed punch biopsies on days 7, 14, 21, and 29. Notably, the correlations for skatole were consistently significant (*p* < 0.05) and positive, except for the correlation observed on day 7 (*p* = 0.14). In contrast, no significant correlations were observed for indole.

## 4. Discussion

One of the most important drug-metabolizing enzyme families in humans and animals comprises a group of hemoproteins, known as cytochrome P450 (CYP), which play a pivotal role in the oxidative metabolism of phase I [34]. Previous investigations have explored the potential of various compounds, aiming to discern their capacity to influence CYP gene expression. Distributed across various tissues, notably in the liver, small intestine, skin, and lungs, these enzymes play a pivotal role in the conversion of both endogenous and exogenous substances, encompassing compounds such as skatole and indole [10,35]. The intricate relationship between skatole production in the hindgut and its subsequent accumulation in adipose tissue remains not fully understood, despite well-documented evidence highlighting the crucial role of hepatic clearance through P(450) CYP in diminishing skatole concentrations in the bloodstream [36]. This hepatic clearance mechanism thereby restricts the accumulation of skatole in adipose tissue. As skatole significantly contributes to boar taint, it is important in the production of meat products and consequently influences their palatability for consumers. Effective strategies to enhance the activity of CYP2E1 and CYP2A enzymes through feed compounds are currently under intensive investigation. The pharmaceutical and chemical properties of dried chicory root [10], as well as those of hydrolysable tannins [15], have demonstrated significant potential to enhance hepatic clearance, characterized by increased enzymatic activity and reduced tissue deposition of skatole.

This study specifically investigated the influence of GEO, OEO, and SC supplementation, focusing on their impact on the CYP2 family, primarily on CYP2E1 and CYP2A.

Oregano is a dietary additive commonly used in various animal production systems [37] due to its known antimicrobial effect and the resulting improved health and performance of growing animals. It is assumed that its chemical properties enable modulations of the gut microbiome and the immune system in piglets [38]. However, there is relatively limited knowledge regarding its effects on hepatic P(450) CYP. Nguyen et al. [14] have demonstrated the effects of functional foods on human P450(CYP) and revealed an inhibitory role of oregano on CYP3A5 and CYP3A7 [39], aligning with the observations by Foster et al. [39]. However, neither of these studies investigated the effects of oregano on CYP2E1 or CYP2A [38]. The present study found no discernible effect of OEO on hepatic CYP activity, nor on skatole or indole concentrations in blood plasma. Consequently, we conclude that OEO does not exert an influence on porcine CYP2E1 or CYP2A.

As reviewed by Nowak et al. [17], a considerable number of studies in the literature have illustrated the health-promoting and beneficial effects of *Schisandra chinensis* on health parameters [40]. The effects of different *Schisandra chinensis* lignans (either singly or in combination) on hepatic enzyme activity have been widely investigated in rodent models [20,40,41,42,43], yet not in adult pigs until now.

Our study focused on enzyme activity, which results from both gene expression and post-translational modifications. The efficiency of these enzymes can be partially estimated by changes in skatole concentrations in blood plasma. Hepatic CYP2E1 and CYP2A activities are important determinants of skatole clearance in pigs and represent major targets for intervention. Our study demonstrated that a long-term treatment of pigs with SC does not lead to reduced skatole accumulation in adipose tissue or enhanced enzymatic activities. Instead, it results in an increase in skatole in blood.

In contrast to studies in rodents, our results suggest that SC has no conclusive impact on porcine CYP2E1 or CYP2A. The investigations of Su et al. [22] in rats reported an enhanced CYP2E1 expression after one single dose of SC extract, whereas multiple doses (14-day supplementation) led to diminished gene expression [43]. A comparable biphasic response to SC lignans has been described by Lai et al. for CYP3A in rats [43], contrasting with the lack of significant changes in hepatic CYP2E1 and CYP2A activity observed in the present study. Nevertheless, we cannot rule out that other factors, particularly complex regulatory processes, might have impeded the translation of higher CYP-mRNA expression into active enzymes [44,45]. Alternatively, disparities between rodent and pig models, such as differences in administered amounts or a potential lack of susceptibility, might have contributed to the observed distinctions.

Notably, researchers utilizing rodent models have administered widely varying concentrations: 75 mg/kg/d [21], 400 mg/kg/d [46], 800 mg/kg/d [21], and 1500 mg/kg/d [20]. This study employed SC concentrations comparable to those used in the investigation of Jin et al. [47]. Given our results and the absence of an increase in CYP activity, we cannot exclude the possibility that the detected rise in skatole in plasma might in part be attributed to the long-term feed with a high purine content in the standard diet, a factor known to favor skatole production in pigs. Since SC extracts are produced from raw plant material, many factors are involved in the reproduction of the desired bioactive activity [48]. Nevertheless, the majority of studies have not specified the parts of the plant utilized or included. This further complicates the replication and extrapolation of results from rodent models to our study in pigs.

The consequences of garlic consumption for CYP2E1 have been intensively studied in rodents and humans [23,47,49]. In these species, it has been demonstrated that garlic induces an irreversible inhibition of the CYP2E1 enzyme, mediated by diallyl sulphide (DAS). Previous studies on the influence of garlic on pigs focused only on growth improvement [50], not on an impact on P(450) CYP’s breakdown after the administration of garlic or garlic derivates. Leong et al. [25] investigated whether a 57-day supplementation of GEO would lead to garlic-flavored meat and reported skatole levels exceeding 1000 ng/g fat. The authors suggested that this was due to an inhibition of CYP2E1 by garlic ingredients. The current study used the same concentration of GEO (0.2%) in the feed and also observed an enhanced skatole accumulation in adipose tissue, but was unable to determine a significant decline in CYP2E1 activity.

Zheng et al. [23] performed a similar experiment with mice and concluded that prolonged treatment resulted in an adaption of CYP2E1 activity. The authors could provoke a substantial reduction after just one single dose of garlic oil and observed a less strong inhibition of CYP2E1 activity after 60 days. Our study did not assess the explicit CYP activity but observed congruent effects by monitoring the strong CYP2E1-dependent skatole levels in plasma. Eight hours after the first single dose of GEO, the blood levels of skatole showed a substantial increase. Surprisingly, and in contrast to the previously mentioned authors, we were not able to detect a significant decline in CYP2E1 activity, except for CYP2A at the end of the experiment. In five out of six animals of the GEO treatment group, the CYP2A activity was lower than in 80% of all the other animals, irrespective of treatment group. However, one animal with high CYP2A activity and nearly unchanged skatole levels in plasma at the end of the treatment period masked the results.

The significant long-term reduction in CPY2A activity, as well as the short-term reaction of skatole levels in the blood to GEO, may strengthen the assumption of a modified hepatic metabolism due to GEO supplementation in pigs. This is further supported by the significant increase in accumulated skatole in adipose tissue and, additionally, may be derived from the long-time course of skatole and indole in the plasma, indicating irregular periodical oscillations.

Most of the studies investigating CYP-mediated influences on boar taint compounds focus on 3-methylindole in pigs but not on indole. Although the degradation of indole seemed not to have been affected by the different treatments in this study, indole may also be sensitive to porcine CYP activity [35] (Appendix B, Figure 1b). This became particularly noticeable as indole in the plasma seemed to be more vulnerable to CYP interference. This strengthens the hypothesis of endogenous adaption, evident from its absence in other treatments. It may signify hepatic compensation in response to exogenous influences affecting CYP, potentially explaining the observed absence of breakdown. Considering the high variability in enzymatic activity and peripheral skatole concentrations, the question arises whether GEO can be used as a tool to characterize the susceptibility of the hepatic metabolism in a genotype, resulting in high skatole values in blood and fat following adverse environmental conditions such as stress [29].

The heterogeneous reaction of hepatic enzymes may be partly explained by variations among individuals, giving the impression that certain animals appear to be non-responders. However, the non-responder animal clearly reacted with increasing skatole levels in its blood during the first 48 h of GEO treatment, but these returned to pre-treatment values during the two-week treatment period. This anomaly might have been caused by genetic polymorphisms within the gene coding for CYP enzymes, as described by Mörlein et al. [51] and Cederbaum [52]. Although genetic variations within or between different breeds of pigs are well documented, the present study showed a considerably wide range of skatole concentrations both in blood and back fat, suggesting the importance of differences in CYP-coding genes. This assumption is supported by the investigations of Rowe et al. [53], which showed that variations in skatole levels have a highly significant association with SNPs of CYP2E1 and consequently might explain the varying accumulation of skatole in adipose tissue, even given a close relationship status. The present data may indicate the genetic difference which, according to Terner et al. [54], is required to establish molecular markers for breeding programs to reduce skatole accumulation in fat and, consequently, influence the levels of boar taint. Moreover, this study highlights the potential to intervene in CYP-mediated skatole degradation, providing an opportunity to conduct challenge tests with short-term GEO application to screen for genotypes with a favored hepatic metabolism at a relatively low cost.

## 5. Conclusions

Our findings suggest that GEO influences the hepatic metabolism of skatole in pigs, while SC and OEO exhibit no significant effects. In the case of GEO, the data clearly demonstrate that a time frame of 24 h is sufficient to increase skatole concentrations in the plasma, indicating the time frame required for the plant extract to interact with hepatic enzymes. However, the dietary additives SC and OEO were unable to induce higher CYP activity in pigs during a 14-day treatment and thus lack the capacity to influence the accumulation of skatole in adipose tissue of the back.

Furthermore, our investigation underscores the potential and efficiency of plant additives due to their rapid mode of action. This study emphasizes the importance of further research on potential dietary additives that might interfere with CYP regulation and thus modulate the degradation of skatole.

## Figures and Tables

**Table 1:** Skatole concentrations in plasma (ng/mL) and back fat (ng/g) at different time points after garlic essential oil (GEO) supplementation (*n* = 6).

	Skatole Concentration						
	Blood Plasma (ng/mL)					Adipose Tissue (mg/g)	
**Animal**	**0 h**	**8 h**	24 h	32 h	48 h	0 h	360 h
**1**	1.60	1.97	4.14	3.89	5.27	75.5	313.3
**2**	1.46	2.22	5.89	3.55	5.19	38.3	164.3
**3**	0.65	1.08	1.82	1.49	1.96	14.0	52.4
**4**	0.98	1.76	3.74	2.91	4.10	30.4	100.4
**5**	0.88	1.33	2.20	2.04	2.72	14.7	39.8
**6**	4.61	4.83	6.17	5.85	6.79	92.3	197.2
**Mean**	1.69 ^a^	2.20 ^b^	3.99 ^c^	3.29 ^d^	4.34 ^c^	44.2 ^a^	144.4 ^b^

Skatole analyses at the different time points coincide with the feeding and correspond to a feeding regimen of twice a day; blood sampling was conducted during the feeding; 0 h = blood sampling occurred immediately before the supplementation of GEO. Different superscripts indicate significant differences (*p* < 0.05 is considered significant in comparison to CON; paired *t*-test).

**Figure A1:**
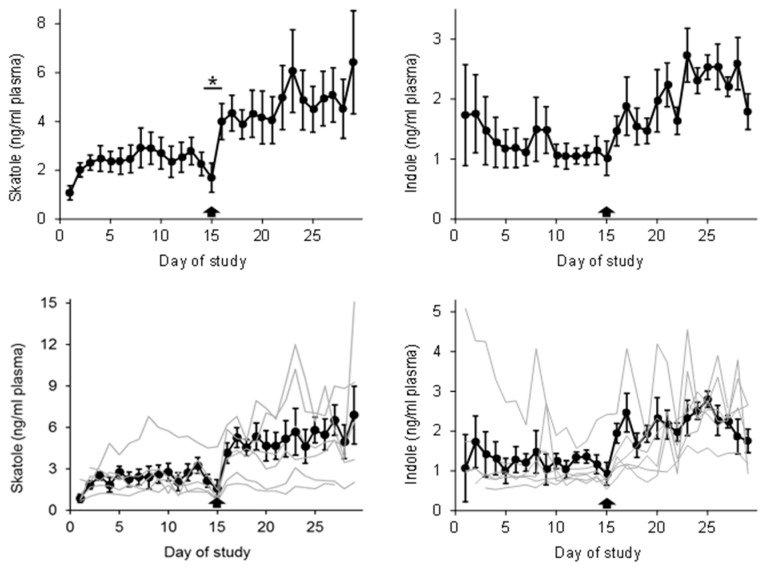
Means ± SEMs pf plasma skatole and indole concentrations of animals treated with garlic essential oil (GEO) (*n* = 6); the arrows indicate the starting point of treatment with GEO; the grey lines are the individual plasma concentrations of the animals; statistics: days 15 and 16: paired *t*-test, * *p* < 0.05 is considered significant in comparison to CON.

## Data Availability

The data presented in this study are available on request from the corresponding author.

## Appendix A

**Table A1:** Analysis of the standard diet.

Analysis of the Standard Diet	g/kg DM
Dry matter	87.5–88.5
Crude ash	3.9–4.1
Crude protein	14.9–16.0
Crude fiber	3.3–3.4
Crude fat	1.9–2.0
Starch	51.2–53.1
MJ/kg	14.0–14.2
**Composition of the Standard Diet**	%
Barley	23.6–23.8
Soy meal	9.3
Triticale	11.9–12.0
Wheat	12.3
Mineral premix	2.7
Soy oil	1.6
Corn starch	26.2–26.3
Brewer’s yeast *	12.1
**Dietary Additives**	%
DOSTO^®^ Konzentrat 500	0.3
*Schisandra* extract	0.3
Garlic essential oil	0.2

* Yeast, MNr. 20102, Leiber GmbH, Bramsche, Germany.
